# Causal insights into gestational diabetes mellitus

**DOI:** 10.3389/fendo.2025.1627919

**Published:** 2025-09-19

**Authors:** Sheresh Zahoor, Anthony C. Constantinou, Fiona O’Halloran, Louise O’Mahony, Mairead O’Riordan, Oratile Kgosidialwa, Linda Culliney, Mohammed Said Alhajri, Mohammed Hasanuzzaman

**Affiliations:** ^1^ Department of Computer Science, Munster Technological University, Cork, Ireland; ^2^ Bayesian Artificial Intelligence Research Lab, Machine Intelligence and Decision Systems (MInDS) Group, Queen Mary University of London (QMUL), London, United Kingdom; ^3^ Department of Biological Sciences, Munster Technological University, Cork, Ireland; ^4^ Cork University Hospital, Cork, Ireland; ^5^ Queen’s University Belfast, School of Electronics, Electrical Engineering and Computer Science (EEECS), Belfast, United Kingdom

**Keywords:** gestational diabetes, Causal Bayesian Networks, causal discovery, interventions, healthcare

## Abstract

**Introduction:**

Gestational diabetes mellitus (GDM), defined by the onset of hyperglycaemia during pregnancy, remains the most prevalent metabolic complication in pregnancy. It is associated with increased risks of adverse maternal, neonatal, and long-term metabolic outcomes. This study aimed to identify potential causal relationships within clinical data on GDM that could support more targeted and effective interventions.

**Methods:**

A clinically curated dataset of patients diagnosed with GDM at a major Irish maternity hospital was analysed, covering the study periods 2014–2016 and 2020. A knowledge graph was constructed by integrating clinical expertise, established literature, and insights generated using the GPT-4 large language model. To complement this, 20 structure learning algorithms were applied to independently infer Causal Bayesian Networks (CBNs). A model-averaging approach was then used to generate a consensus-based causal structure to account for variability across individual models.

**Results:**

The integrative model produced a more stable representation of underlying relationships and yielded quantifiable insights to support clinical decision-making. Clinicians involved in the study reported improved confidence in patient care strategies due to the ability to quantify these relationships, facilitating more personalised, evidence-based practice. Key findings from the model-averaged CBN highlighted critical pathways in GDM management, such as the influence of birth weight on neonatal intensive care unit (NICU) admissions and the impact of dietary intervention on maternal glucose regulation. Sensitivity analysis confirmed birth weight, gestational age at delivery, and mode of delivery as major determinants of maternal and neonatal outcomes. Non-modifiable factors, including a history of multiple pregnancies and prior GDM, also contributed to risk stratification.

**Discussion:**

This study applied structure learning techniques to observational clinical data to identify clinically relevant relationships. The resulting insights provide a basis for generating hypotheses that could refine intervention strategies and improve patient outcomes in GDM care.

## 1 Introduction

Gestational diabetes mellitus (GDM) is a prevalent complication of pregnancy, characterised by the onset of glucose intolerance during gestation (1). A recent meta-analysis estimated that the global standardised prevalence of GDM is approximately 14.0% (95% confidence interval: 13.97–14.04%) (2), although rates vary considerably amongst populations. These differences largely stem from the diverse screening procedures and diagnostic criteria employed internationally (3–5). For over a decade, research has consistently shown that effective management of GDM not only improves pregnancy outcomes but also reduces the incidence of gestational hypertension and pre-eclampsia (6, 7). While GDM is generally regarded as an acute condition associated with short-term complications, it may develop at any stage of pregnancy, most commonly during the second trimester and typically resolves after childbirth.

Numerous factors increase the risk of developing GDM, with a family history of diabetes and being overweight or obese playing significant roles (8). Women diagnosed with GDM face a higher likelihood of complications during pregnancy, including polyhydramnios, pre-eclampsia, and the need for a caesarean delivery (9). Moreover, GDM carries an elevated lifetime risk of progressing to type 2 diabetes, with nearly half of affected women developing abnormal glucose metabolism within 10 years of the pregnancy (10, 11). For instance, the HAPO Follow-up Study found that 52% of women with GDM later exhibited abnormal glucose metabolism, compared to just 20% of those with normal glucose tolerance after 10–14 years (11). In addition to a higher risk of diabetes, a history of GDM is associated with several cardiovascular risk factors, such as obesity, hypertension and dyslipidaemia (12), which have in turn been linked to increased rates of ischaemic heart disease in large observational studies (12, 13).

GDM diagnosis is linked to increased morbidities for both the mother and infant in the short and long term. Infants born to mothers with GDM face risks such as accelerated fetal growth, macrosomia (birth weight > 4500g), and preterm delivery (14, 15). Macrosomia, in turn can lead to birth trauma, including shoulder dystocia, nerve palsy, and fractures (16). Additional neonatal complications associated with GDM include respiratory distress syndrome, neonatal hypoglycemia, hyperbilirubinemia, polycythemia, and hypocalcemia (17). Beyond these immediate concerns, emerging research suggests that the altered intrauterine environment in GDM may predispose affected infants to long-term health issues. They have an increased risk of developing obesity, cardiovascular disease, and type 2 diabetes mellitus later in life (18). This may be due to early metabolic programming triggered by maternal hyperglycaemia, which can affect pancreatic beta-cell function and insulin sensitivity in the neonate. Consequently, these infants may require careful postnatal monitoring and early interventions aimed at reducing their long-term risk of chronic metabolic disorders.

Several studies suggest that early measures to maintain normal blood glucose levels can help prevent complications for both mother and child (6, 19). In line with this, a recent systematic review and meta-analysis (20) demonstrated that interventions, ranging from dietary modifications and physical activity programmes to combined approaches and pharmacological treatments such as metformin and myoinositol, can substantially reduce the incidence of GDM. The analysis also highlighted that intervention effectiveness is not uniform across all individuals. For example, physical activity programmes delivered in group settings or within healthcare facilities tend to yield more significant benefits than those conducted individually or through community-based approaches. While diet-only interventions perform consistently across various settings, combined strategies appear to be particularly effective in low to middle-income countries compared with higher-income regions. These findings highlight the importance of tailoring intervention strategies to the unique characteristics of the target population and local context, thereby maximising their potential to prevent GDM and its related complications.

While current intervention strategies show promise, a comprehensive understanding of how demographic characteristics, clinical markers, and lifestyle factors interact to shape GDM risk and disease trajectories remains elusive. This knowledge gap hampers the development of targeted interventions and personalised care strategies for both the prevention and management of GDM. To bridge this gap, the study employed a multifaceted approach using Causal Bayesian Networks (CBNs) to explore potential points of influence within the clinical care pathway, with the aim of supporting the development of more tailored strategies to improve maternal and neonatal health outcomes. Unlike traditional associative models, CBNs facilitate causal reasoning by modelling andevaluating the conditional dependencies and potential pathways that may underlie GDM progression, while acknowledging the limitations inherent in observational data.

CBNs are probabilistic graphical models that encode the relationships amongst variables, offering an interpretable framework to explore complex interactions in medical research. Beyond identifying correlations, this approach aims to distinguish between potential direct and indirect effects of risk factors on health outcomes and supports interventional analysis under specific assumptions, which are essential for causal inference. The simulation of hypothetical interventions within these learned structures allows researchers to estimate potential effects prior to real-world implementation. In the context of GDM, this methodology provided a principled means to investigate potential intervention targets, assess their likely downstream effects, and support the design of more tailored strategies to optimise maternal and neonatal health outcomes.

This study sought to address a clinically relevant yet underexplored question: how do modifiable and non-modifiable maternal factors interact to influence both maternal and neonatal outcomes in the context of GDM, and how might these interactions inform the timing and nature of targeted interventions? Conventional approaches, such as logistic regression or clinical risk scoring systems, often rely on predefined assumptions about variable relationships and may not fully capture the complexity of interdependent clinical factors. In contrast, Bayesian network modelling provides a structured, interpretable framework for identifying potential causal pathways and evaluating how changes in one factor may influence others within the system. This approach offers a complementary perspective to standard methods by supporting more nuanced, data-informed decision-making in GDM care.

To apply these concepts, this study utilised a novel dataset from Cork University Maternity Hospital (CUMH), a large maternity unit in the South of Ireland. Through causal discovery analysis, a CBN was constructed to investigate the complex relationships between various factors and GDM outcomes. This approach not only facilitated the identification and modelling of underlying causal pathways but also provided a structured framework for deepening our understanding of the factors that influence GDM, paving the way for more effective and tailored interventions.

The key contributions of this research are:

1. Developed a comprehensive causal graph for GDM by integrating relevant data variables. To accelerate up the process, the initial graph was generated using GPT-4 as a preliminary domain expert and subsequently refined through input from clinical experts, ensuring that key relationships and attributes pertinent to the disease were accurately captured.
2. Identified causal pathways influencing maternal and neonatal outcomes by applying 20 structure learning algorithms, thereby facilitating an unbiased exploration of the underlying causal mechanisms.
3. Evaluated the stability and reliability of these causal pathways across multiple algorithms to ensure the generalisability and consistency of the findings.
4. Constructed a model-averaged CBN that synthesises insights from various algorithms, thereby reducing biases and enhancing the robustness of the inferred causal structure.
5. Validated the causal model through comparison with clinical expert insights, and investigated the alignment of algorithmic outputs with domain knowledge.
6. Demonstrated the practical utility of the approach by employing CBNs to simulate and evaluate the potential impacts of hypothetical interventions, thereby providing evidencebased insights to inform targeted strategies for GDM prevention and management.

This research aimed to enhance the understanding of GDM and contribute to the development of targeted, evidence-based interventions to improve maternal and neonatal outcomes. The subsequent sections provide a detailed description of the study population, outline the methodology employed, and present the findings of the analysis. This is followed by a discussion of the clinical implications of the results.

## 2 Materials and methods

### 2.1 Study population

This study utilised a novel and specialised clinical dataset comprising records from 1,808 patients diagnosed with GDM at CUMH, one of Ireland’s largest maternity units. Of these, 1,488 patients had a documented mode of delivery, indicating completed pregnancies with delivery outcomes. In contrast to broader perinatal datasets that include a heterogeneous mix of maternal health conditions, this dataset was exclusively focused on GDM, allowing for a more nuanced and condition-specific analysis. Thistargeted design facilitated a detailed examination of factors influencing both the management and outcomes of GDM within a real-world clinical setting.

The selection of variables was guided by clinicians and healthcare professionals with expertise in maternal–fetal medicine and endocrinology at CUMH. Their involvement was instrumental in identifying variables of clinical relevance, ensuring that the dataset captured the complexities and priorities associated with GDM care. This collaborative, expert-led approach underpinned the study’s clinical utility and provided a robust foundation for generating evidence-based, actionable insights. A comprehensive list of the included variables, along with the rationale for their selection, is presented in **Table 1**.

**Table 1:** List of variables analysed in the study, including demographic, clinical, and metabolic parameters, along with rationale and quantisation method.

Variable	Rationale for inclusion	Quantisation
Age	Advancing maternal age is a well-established risk factor for GDM.	≤ 24 y = 1; 25–34 y = 2; ≥ 35 y = 3; missing = 99
Ethnicity	Certain ethnic groups have a higher prevalence of GDM.	Irish/Irish Traveller = 1; White = 2; Black/African = 3; Asian = 4; Other = 5; missing = 99
Occupation	Proxy for socioeconomic status.	Managers = 1; Professionals = 2; Technicians = 3; Support = 4; Manual = 5; Non-workforce = 6; missing = 99
Marital Status	Proxy for social support and economic stability.	Married = 1; Single = 2; Separated/Widowed/Divorced = 3; Missing = 99
Patient Type	Differentiates public vs. private healthcare settings.	Public = 0; Private= 1; missing =99
Medical History	Includes pre-existing cardiovascular/metabolic conditions.	No =0; Yes =1; missing =99
Diabetes	Identifies pre-existing diabetes.	No =0; Yes =1; missing =99
Thyroid	Thyroid disorders linked to insulin resistance.	No =0; Yes =1; missing =99
Hypertension	Known risk factor for adverse pregnancy outcomes.	No =0; Yes =1; missing =99
Depression	Mental health influencing adherence to management.	No =0; Yes =1; missing =99
Previous Bariatric Surgery	Alters metabolic function and insulin sensitivity.	No =0; Yes =1; missing =99
Smoking/Alcohol Intake	Modifiable lifestyle factors.	Smoking -Never smoked = 0; Smokes during pregnancy = 1; Smoked before pregnancy = 2; Former smoker = 3; Missing = 99/None = 0; Alcohol -Drinking in Pregnancy = 1; Not in Pregnancy = 2; Missing/Other = 99
Gravida/Parity	Cumulative pregnancies affect GDM risk.	Nulligravida = 0; Primigravida = 1; Multigravida = 2; Missing/Other = 99/Nulliparity = 0; Low Multiparity (1–2 previous pregnancies) = 1; Grand Multiparity (≥ 3 previous pregnancies) = 2; Missing/Other = 99
BMI Category	Strong predictor of GDM.	Underweight = 1; Healthy = 2; Overweight = 3; Obese = 4; Missing/Other = 99
Change in Weight	Excessive gestational weight gain linked to poor outcomes.	Low = 0, Normal = 1, High = 2 (BMI-specific IOM gestational weight gain ranges), Missing = 99
Folic Acid Intake	Impact on fetal development and GDM prevention.	No =0; Yes =1; missing =99
Multivitamin Use	Role of micronutrient supplementation.	No =0; Yes =1; missing =99
Past History of GDM	Strong predictor of recurrence.	No =0; Yes =1; missing =99)
History of PCOS	Linked to insulin resistance.	No =0; Yes =1; missing =99
History of Large Baby (>4.5kg)	Indicator of future GDM risk.	No =0; Yes =1; missing =99
Family History of Diabetes	Genetic predisposition to GDM.	No =0; Yes =1; missing =99
Previous Obstetric History	Previous pregnancy complications.	No =0; Yes =1; missing =99
Fertility Treatment	Higher incidence of GDM with ART.	No =0; Yes =1; missing =99
Gestational Age at Diagnosis	Early diagnosis indicates higher risk.	First trimester = 1, Second trimester = 2, Third trimester = 3, Missing/Unknown = 99
GTT Location	Differences in diagnostic setting.	CUH = 1, GP = 2, Private Rooms = 3, Other = 4, Missing/Unknown = 99
GTT Result	Fasting and 2-hour postprandial glucose.	Normal = 1, At risk/High risk = 2, Missing/Unknown = 99
HbA1c at Diagnosis	Alternative diagnostic marker.	<35 = 1, 35–40 = 2, 41–47 = 3, >48 = 4, Missing = 99
Scan at Diagnosis	Fetal growth parameters.	Growth- Very Low = 0, Low = 1, Normal = 2, High = 3, Very High = 4, Missing = 99/AC- Small = 0, Appropriate = 1, Large = 2, Missing = 99
Diet-Only Treatment	Managed without pharmacological therapy.	No =0; Yes =1; missing =99
Referral to Endocrinology	Specialist involvement.	No =0; Yes =1; missing =99
Metformin Use	Effectiveness in GDM treatment.	No =0; Yes =1; missing =99
Insulin Use	Therapy initiation and impact.	No =0; Yes =1; missing =99
Pregnancy Complications	Includes pre-eclampsia, polyhydramnios, etc.	No =0; Yes =1; missing =99
Gestation at Delivery	Preterm birth rates.	First trimester = 1, Second trimester = 2, Third trimester = 3, Missing/Unknown = 99
Live Infant	Perinatal survival rates.	No =0; Yes =1; missing =99
Delivery Mode	Mode of birth.	Vaginal =0; Operative =1; Instrumental delivery =2; missing =99)
Maternal Trauma	Postpartum haemorrhage, trauma, etc.	No =0; Yes =1; missing =99
Infant Outcomes	NICU admission, macrosomia, etc.	Birth Weight -Macrosomic = 1, Normal = 2, LBW = 3, VLBW = 4, ELBW = 5, Missing = 99/NICU admission- Yes = 1, No = 2, Missing = 99
Breastfeeding on Discharge	Initiation of breastfeeding.	No =0; Yes =1; missing =99
Postnatal Glucose Tolerance Test	Postpartum glucose control.	No =0; Yes =1; missing =99
Other Postnatal Tests	Fasting glucose, HbA1c post-pregnancy.	Normal = 1, At risk/High risk = 2, Missing/Unknown = 99

### 2.2 Data preprocessing

This study was approved by the Clinical Research Ethics Committee of the Cork Teaching Hospitals (CREC Review Reference Number: ECM 4 (o) 06/02/2024), ensuring compliance with data confidentiality standards and ethical guidelines for patient information. Maternal records of women diagnosed with GDM who delivered at CUMH across four years (2014, 2015, 2016, and 2020) were used in this research. The years 2014–2016 and 2020 were selected based on the availability of complete electronic health records at the time of data extraction. Records from 2017 to 2019 were not included, as they had not yet been fully integrated into the hospital’s clinical information system and were therefore inaccessible. As the analysis did not model temporal or cohort effects explicitly, this gap is not expected to have materially influenced the structure of the inferred CBN. While the inclusion of data from 2020 may have introduced some indirect effects related to the COVID-19 pandemic—such as changes in care delivery or patient behaviour, no COVID-specific variables were available, and the potential impact of the pandemic could not be separately assessed.

The dataset, initially provided as Excel spreadsheets with each year represented on a separate sheet, included both categorical and continuous variables. Prior to causal structure learning, preprocessing steps were undertaken to accommodate the requirements of most structure learning algorithms, which typically operate on discretised data. Continuous variables, such as maternal age, BMI, blood glucose levels, and gestational timing—were discretised using clinically meaningful thresholds where available (e.g., BMI categories), or through data-driven binning informed by quantiles and expert clinical input. This strategy was chosen to preserve interpretability while minimising the risk of information loss or distortion often associated with arbitrary binning.

While discretisation can reduce sensitivity to subtle trends within the data, it enabled consistent modelling across heterogeneous variable types and avoided reliance on strong distributional assumptions. Any inconsistencies in variable categorisation were addressed through iterative discussions with the clinical team, who acted as domain experts and primary dataset curators. These collaborative steps ensured that the final dataset maintained clinical relevance while remaining suitable for robust structure learning. Also, data on pre- and post-pregnancy diet and physical activity were not consistently or systematically recorded and were therefore excluded from the analysis. Their omission may limit insights into lifestyle-related influences on maternal and neonatal outcomes.

As is common with clinical datasets, missing values posed a challenge, particularly in key variables, potentially affecting the completeness and robustness of the analyses. These issues were addressed through rigorous data cleaning and validation, with selective exclusion applied where missingness was substantial. This process aimed to minimise the impact of missing data, thereby improving the dataset’s reliability for subsequent modelling and analysis.

#### 2.2.1 Missing values

Structure learning algorithms typically require complete datasets to function optimally, including those employed in this study. However, the dataset contained instances of missing values, arising from incomplete medical records, inconsistent data entry, or gaps in patient follow-up, challenges frequently encountered in clinical research.

**Figure 1:**
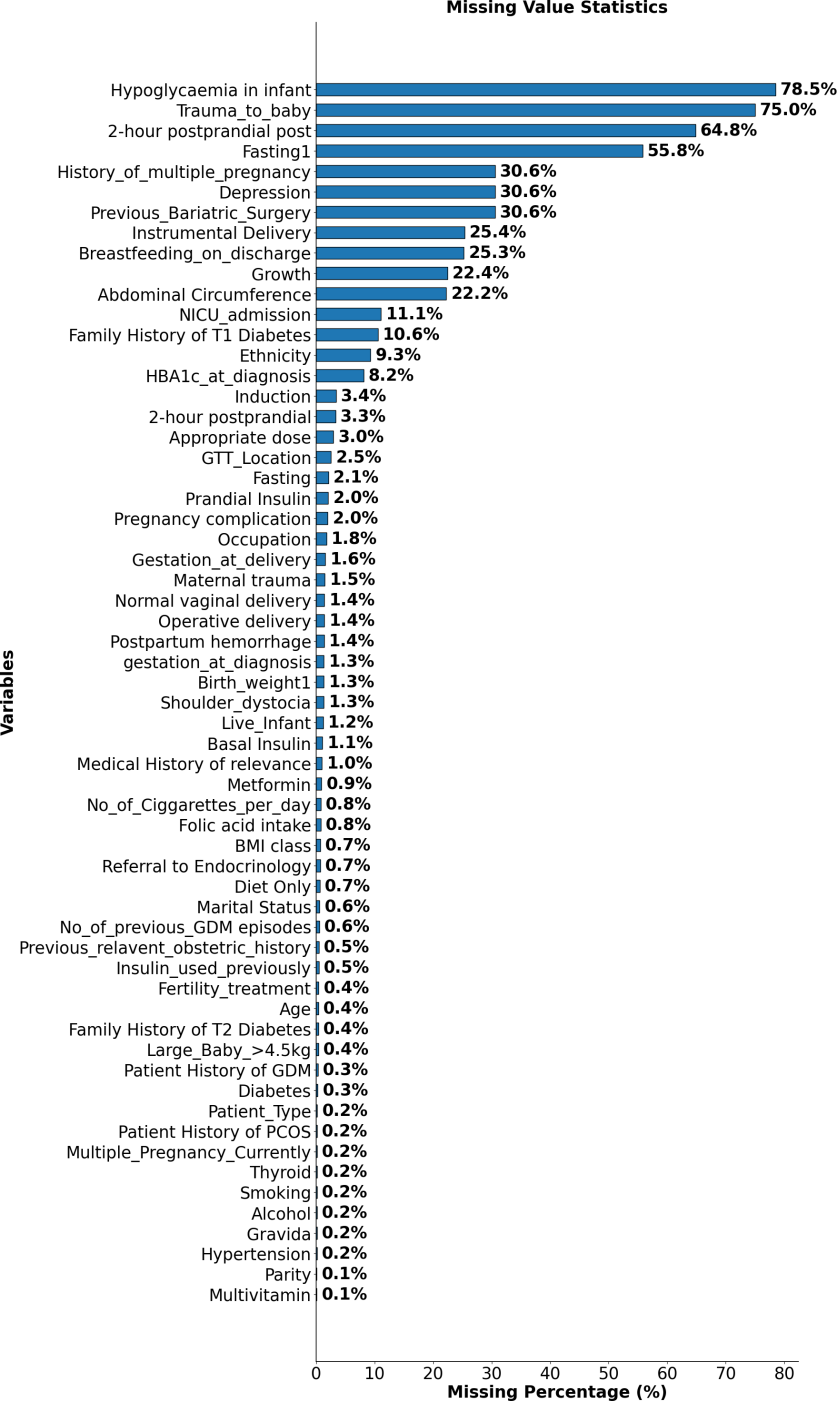
Proportion of missing data across variables in the GDM dataset. This figure highlights variables with the highest missing values, such as Hypoglycaemia in Infant, Trauma to Baby, and 2-hour postprandial glucose after the delivery.

**Figure 1** illustrates the percentage of missing values for each variable in the GDM dataset, identifying those most affected by incomplete data. Notably, variables such as “Hypoglycaemia in Infant”, “Trauma to Baby”, and “2HRPPpost (2-hour postprandial glucose after the delivery)” exhibit over 70% missing values. Additionally, variables like “Previous Bariatric Surgery” and “Depression” also show a significant degree of missing data. The prevalence of missing values in these key variables could impact the strength and interpretability of subsequent analyses.

To address this challenge, missing values were not excluded but instead categorised under a distinct label, “99”. This approach, informed by prior literature (21), enabled the retention of valuable records without assumingthat missingness occurred completely at random. In many variables, missing data rates exceeded 70%, suggesting that the missingness was likely systematic and reflective of real-world clinical decision-making or documentation patterns. In such cases, imputation methods risk introducing misleading values and can reduce the accuracy of structure learning. By treating missingness as a separate state, the model preserves potentially informative signals while minimising information loss and analytical bias.

While this method ensured the dataset remained as comprehensive as possible, it is important to acknowledge that treating missing values as a separate category may affect the interpretation of certain relationships, since the missingness in a variable may depend on the values of some other variable. Nonetheless, this approach enabled the inclusion of all available data while reducing the bias that might have resulted from excluding incomplete records or imputing missing values.

#### 2.2.2 Data analysis and visualisation

**Figure 2:**
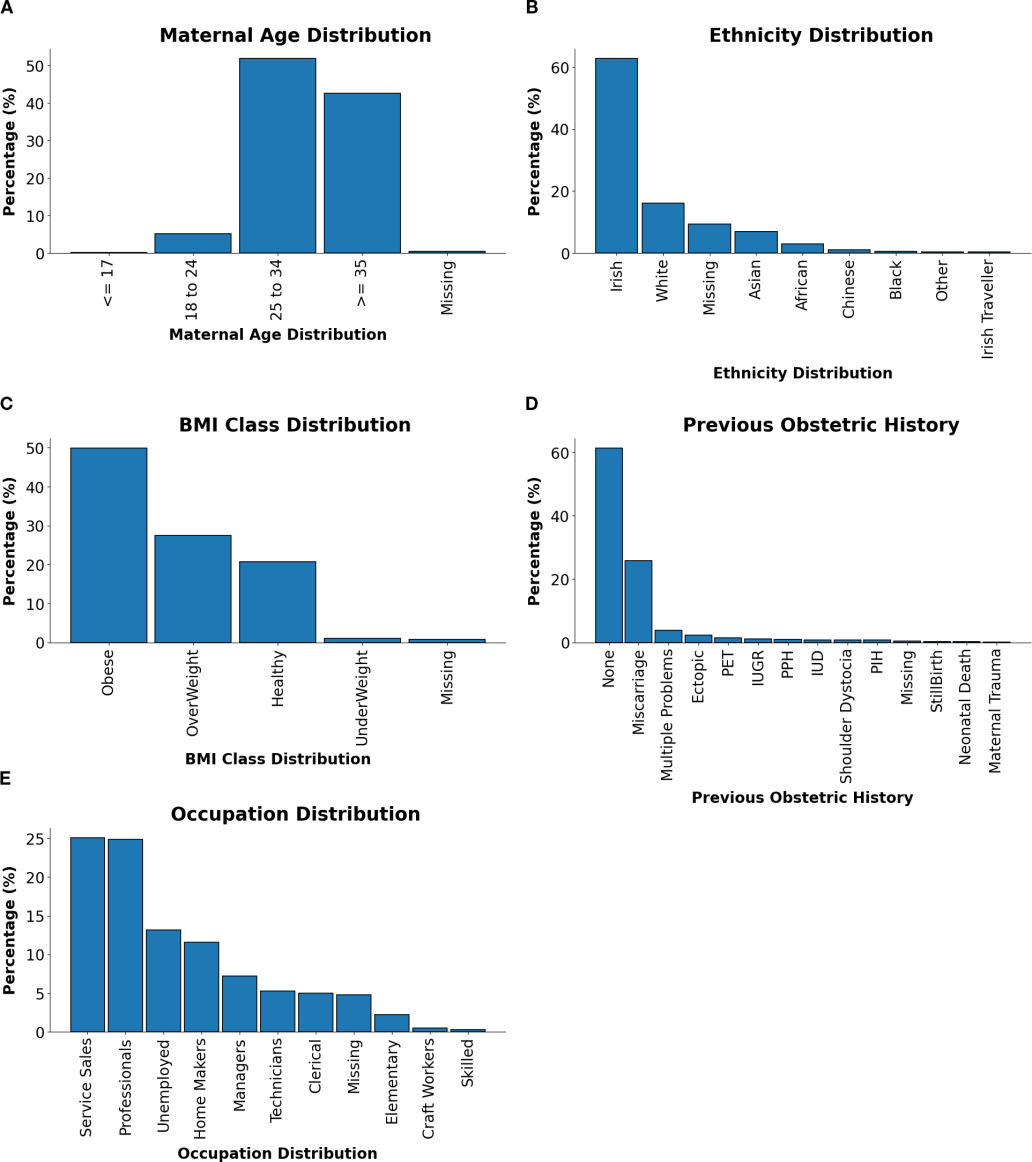
Distributions of maternal characteristics and clinical factors in GDM-positive patients. **(A)** Maternal age distribution. **(B)** Ethnicity distribution. **(C)** BMI class distribution. **(D)** Previous relevant obstetric history distribution. **(E)** Occupation distribution.

This section summarises the demographic and clinical characteristics of the GDM cohort, as presented in **Figure 2**, which provides an integrated visualisation of maternal age, ethnicity, BMI class, previous obstetric history, and occupation.

Maternal Age Distribution **Figure 2A** showed that the majority of patients were aged between 25–34 years or ≥35 years, reflecting the heightened risk of GDM with advancing maternal age. This trend was likely due to reduced glucose tolerance and higher prepregnancy BMI observed in older individuals.

Ethnicity Distribution As shown in **Figure 2B**, Irish patients comprised the largest proportion of the cohort, followed by smaller groups of White, Asian, and African backgrounds. This diversity underscored the importance of culturally tailored approaches to GDM management, recognising differences in dietary patterns, health behaviours, and access to healthcare services.

BMI Class Distribution As shown in **Figure 2C**, the BMI distribution was dominated by obese and overweight categories, consistent with the established link between elevated BMI and GDM risk. These findings highlighted the significance of pre-pregnancy weight management and targeted lifestyle interventions as key strategies for GDM prevention.

Previous Relevant Obstetric History **Figure 2D** shows the distribution of previous obstetric history. While a majority of patients reported no prior complications, a notable subset presented with histories of miscarriage, multiple pregnancies, or pre-eclampsia. These patterns emphasised the need for individualised risk assessments and tailored care pathways.

Occupation Distribution The occupational profile, visualised in **Figure 2E**, indicated that Service Sales and Professional occupations were the most common, followed by Homemakers and Unemployed individuals. This range reflected socioeconomic diversity within the cohort and suggested potential influences of occupational status on GDM risk, health literacy, and access to care.

### 2.3 Learning causal Bayesian networks

This study followed a two-step approach to learning CBNs. First, a knowledge-based graph was constructed using outputs from GPT-4, supported by evidence from the empirical literature and refined through clinical expert review. This graph served as a prior conceptual framework grounded in established domain knowledge. Second, a data-driven structure learning approach was implemented, applying 20 algorithms to the dataset to uncover potential causal relationships directly from observed clinical data.

The overall process for CBN construction comprised the following stages:

1. Knowledge-Based Structure Construction: Created an initial causal graph using GPT-4 and the literature, then refined it with clinician expertise.
2. Structure Learning from Data: Applied 20 structure learning algorithms to the dataset to identify causal relationships between variables.
3. Model Averaging: Used model-averaging to combine the outputs of different algorithms into a consensus structure.
4. Comparison with Knowledge-Based Structure: Compared data-driven structures with the knowledge-based graph to assess consistency.
5. Use of the CBN: Employed the knowledge graph and model-averaged graph for causal inference, simulation of interventions, and sensitivity analysis.

#### 2.3.1 Knowledge-based structure construction

In this step, a hybrid approach was employed, combining clinical expertise, literature review, and Large Language Models (LLMs), such as GPT-4, serving as an imperfect domain expert, to construct the initial causal knowledge graph. Recent research has highlighted the potential of LLMs to function as imperfect domain experts capable of identifying causal relationships. For instance, as demonstrated in (22), GPT-4 can infer causal connections from variable labels alone, despite not being explicitly designed for causal reasoning. The results suggested that combining LLMs with causal machine learning (ML) algorithms improves causal discovery but helping algorithms avoiding common-sense mistakes, producing structures that more closely align with expert knowledge.

To leverage this potential, GPT-4 was utilised to infer initial causal relationships among the variables in the dataset. The system was provided only with variable labels, in line with the method described in the cited study, where GPT-4 was prompted to reason about potential causal connections using semantic information encoded in the labels. Specifically, GPT-4 was tasked iteratively with constructing a directed graph, where nodes represented variables and edges denoted potential causal relationships. This approach provides a dataefficient means of generating causal structures by using the reasoning capabilities of LLMs. However, while GPT-4 can propose meaningful causal relationships, grounding these in scientific evidence and clinical knowledge is essential.

The initial graph was refined through a systematic two-step process. While GPT-4 was used to generate an initial causal structure based on variable semantics, this was only the starting point for graph construction. A targeted literature review was conducted not to endorse the suggestions of GPT-4, but to independently validate and contextualise the inferred edges. Specifically, the literature review served two main purposes: (1) to confirm edges supported by empirical evidence, and (2) to identify potentially spurious or weakly supported edges for removal or revision. Relevant studies were identified using structured searches that combined variable names (e.g., “BMI class and Diabetes” or “Smoking and Pregnancy Complications”) with the term “risk factors”. The review focused on research related to gestational diabetes, pregnancy complications, and related metabolic conditions, prioritising empirical studies and reviews published within the last 10 years. For instance, edges like (Smoking, Pregnancy Complications”) with the term “risk factors”. The review focused on research related to gestational diabetes, pregnancy complications, and related metabolic conditions, prioritising empirical studies and reviews published within the last 10 years. For instance, edges like (Smoking, Pregnancy_complication) (23), (BMIclass, Diabetes) (24), and (Ethnicity, Diabetes) (25) were strongly supported by the literature, reinforcing their inclusion in the graph. Conversely, relationships such as (Change_in_Weight, Diabetes) (26) exhibited mixed evidence, with certain studies highlighting potential confounders or context-specific factors. Edges with limited evidence, such as (AC, Growth) (27), were flagged for further clinical review. Eventually, edges with limited or inconclusive evidence were excluded, as they were deemed to likely represent indirect relationships rather than direct causal relationships.

In the second step, the graph was reviewed by clinicians.Two clinical specialists participated in this process. Each expert was provided with the preliminary graph, a list of proposed edges, and a summary of supporting or conflicting literature where available. They independently reviewed the edges, assessing whether each relationship aligned with their clinical knowledge and experience. A follow-up meeting was then conducted to discuss discrepancies and achieve consensus on the final structure.

**Figure 3:**
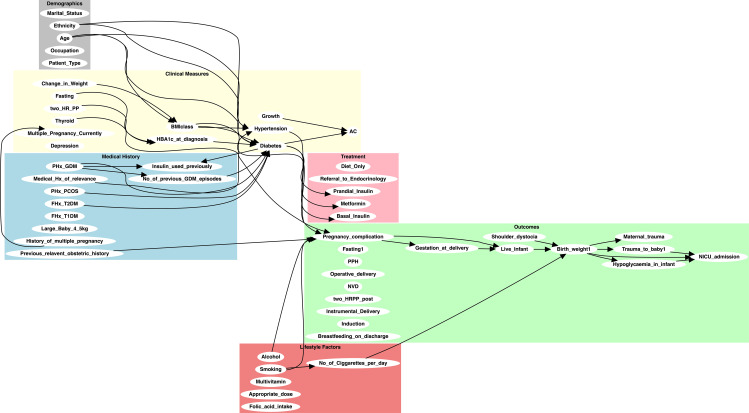
Initial knowledge-based causal graph constructed using GPT-4 and refined through expert clinical review. The graph illustrates the relationships between key variables in the GDM dataset, integrating both domain-specific knowledge and insights inferred by GPT-4.

Their review focused on assessing the clinical plausibility and relevance of the proposed edges, ensuring alignment with current clinical practice and guidelines. Edges were removed if they were considered speculative, redundant, or clinically unjustifiable despite computational support. For example, the edges (Large_Baby_4_5kg, Diabetes) and (Change_in_Weight, Diabetes) were excluded due to concerns about causal direction and confounding. This iterative validation process ensured that the final knowledge graph integrated computational inference, empirical evidence, and clinical expertise, resulting in a structured and contextually grounded representation of potential causal relationships. **Figure 3** presents the refined causal knowledge graph constructed through this hybrid process.

#### 2.3.2 Structure learning from data

Data-driven structure learning techniques were employed to identify potential causal relationships within the dataset. These techniques utilise algorithms to identify conditional independencies and interactions between variables based on observed data, offering a comprehensive view of the underlying relationships and uncovering patterns not immediately evident through traditional analysis.

This study employed a diverse set of 20 structure learning algorithms to comprehensively explore the data (see **Table 2**). These algorithms represent different classes of methodologies and include score-based learning, constraint-based learning, and hybrid approaches, allowing us to capture a broad range of potential causal structures based on varying assumptions and search strategies. Each algorithm offers unique strengths for exploring the complex relationships within the dataset, which was particularly important given the nuanced nature of the maternal and neonatal outcomes under investigation.

**Table 2:** List of structure learning algorithms used in the study, with learning class, reference, and implementation tool.

Algorithm name	Learning class	Reference	Tool used
GES (Greedy Equivalence Search)	Score-based	(28)	Bayesys
HC (Hill Climbing)	Score-based	(28)	Bayesys
TABU	Score-based	(29)	Bayesys
SaiyanH	Hybrid	(30)	Bayesys
MAHC (Max-min Adaptive Hill Climbing)	Score-based	(31)	Bayesys
PC-Stable	Constraint-based	(32)	bnlearn
IAMB (Incremental Association Markov Blanket)	Constraint-based	(33)	bnlearn
InterIAMB (Interleaved IAMB)	Constraint-based	(33, 34)	bnlearn
FastIAMB (Fast IAMB)	Constraint-based	(34)	bnlearn
H2PC (Heuristic Hill Climbing & PC-Stable)	Hybrid	(35)	bnlearn
MMHC (Max-Min Hill Climbing Hybrid)	Hybrid	(36)	bnlearn
RSMAX2	Hybrid	(37)	bnlearn
IAMB-FDR (IAMB with FDR Control)	Constraint-based	(38)	bnlearn
MMPC (Max-Min Parents and Children)	Constraint-based	(39)	bnlearn
GS (Grow-Shrink)	Constraint-based	(40)	bnlearn
IMaGES	Score-based	(41)	Tetrad
FGES (Fast Greedy Equivalence Search)	Score-based	(42)	Tetrad
GIES (Greedy Interventional Equivalence Search)	Score-based	(43)	Causal Discovery Toolbox(Python)
NOTEARS	Score-based	(44)	Causal Discovery Toolbox(Python)
LiNGAM (Linear Non-Gaussian Acyclic Model)	ICA-based	(45)	Tetrad

To apply these algorithms, some commonly used tools were employed: TETRAD by (41), Bayesys by (46), and bnlearn by (47) and Casual Discovery Toolbox (48). These tools were used to apply the 20 algorithms to our dataset, producing a range of graphical structures. Additionally, Bayesys was utilised for model averaging, and also to convert the learnt graphical structures into BN models. These models were loaded into the GeNIe BN software (49) for causal inference and simulation of interventions.

As not all algorithms produce fully directed acyclic graphs (DAGs), particular attention was given to constraint-based methods, which typically output Completed Partially Directed Acyclic Graphs (CPDAGs). To enable the construction of CBNs and supportinterventional analysis, each CPDAG was converted into a DAG by randomly selecting one representative from its Markov equivalence class. While this step introduces some uncertainty in edge orientation, its effects were mitigated by applying model averaging across multiple algorithms, which helped stabilise the final structure and reduce reliance on any single model’s assumptions.

More broadly, most of the structure learning algorithms employed assume causal sufficiency, that is, all common causes of the measured variables are observed. While this assumption may not hold entirely in real-world clinical datasets, efforts were made to reduce the risk of unmeasured confounding by involving clinicians in the variable selection process to ensure comprehensive inclusion of relevant factors. Although latent confounders may still exist, the combined use of diverse algorithms and a model-averaging strategy provided a measure of robustness against spurious or unstable edges arising from hidden variables.

#### 2.3.3 Model averaging

The literature has shown that no single algorithm consistently outperforms others across all scenarios (21, 50, 51), and that the learnt structures are highly sensitive to the choice of algorithm, leading to potential inconsistencies in the resulting graphs. To address this sensitivity, a model averaging approach was employed to produce a single graph that consolidates insights from all 20 structure learning algorithms evaluated in this study. Rather than relying on a single best-performing model, this approach integrates the structural outputs of multiple methods to yield a more stable and comprehensive representation of the underlying causal relationships. The open-source Bayesys tool was used to implement model averaging in an automated and reproducible manner. The averaging process begins by adding edges with the highest counts first, ensuring that the most frequently agreed-upon relationships across algorithms are prioritised. Edges with less agreement may result in conflicting orientations, and in such cases, maintaining acyclicity takes precedence over majority directionality. This helps ensure that the resulting graph remains a valid DAG.

The Bayesys model averaging procedure involves the following steps:

1. Add directed edges to the average graph, starting from highest occurrence;
  a. Skip edge if already added in reverse direction;
  b. Skip edge if it produces a cycle, reverse it and add it to edge-set;
2. Add undirected edges starting from highest occurrence;
  c. Skip edge if already added as directed;
3. Add directed edges found in starting from highest occurrence;
  d. Skip edge if already added as undirected.

A key consideration in the averaging process is determining how many edges to include in the final averaged graph. This requires selecting a cut-off point; e.g., whether to include edges that appear in at least one, two, or more structures. In this study, the average model was found by including edges that appeared in at least four of the 20 learnt structures. An unweighted majority-vote approach was used for model averaging. This method was chosen to avoid bias toward any particular algorithm class, given their diverse assumptions and performance characteristics. While weighted model averaging was not implemented, future work may explore weighting schemes that incorporate algorithm confidence scores or domain-informed priors to further refine edge inclusion.

To ensure acyclicity during model averaging, edges that would have introduced cycles were automatically reversed and included in the final graph, as implemented in the Bayesys tool. While this step maintains the structural integrity of a DAG, it may introduce uncertainty in the directionality of specific edges. These reversals were handled programmatically and not flagged separately in downstream inference. Although not explicitly annotated, we acknowledge that such adjustments could influence causal interpretation and should be considered when assessing the robustness of inferred relationships.

### 2.4 Evaluation

The performance of each algorithm was evaluated using seven distinct criteria:

1. Structural Hamming Distance (SHD) Comparison: The relationship between the learnt graphs and the reference knowledge-based graph was assessed using the SHD score, a graphical metric that calculated the number of differences (i.e., Hamming distance) between two graphs. SHD scores were reported by comparing the graphs learnt by the algorithms to the graph constructed by the clinical expert and GPT-4.
2. Independent Graphical Fragments: The number of independent graphical fragments (disjoint subgraphs) was counted for each algorithm. This criterion was important because information flow between all variables was not possible in structures containing independent fragments, which could limit insights. Given that the dataset contained related variables—aside from a few exceptions such as ethnicity, a high number of disjoint subgraphs indicated a failure to capture important connections.
3. Graph Complexity and Free Parameters: The complexity of each graph was determined by the number of free parameters, representing the additional parameters introduced by each new edge. This provided insight into how complicated the learnt models were.
4. Bayesian Information Criterion (BIC) Score: The BIC score served as a key model selection criterion for comparing the structural performance of different algorithms. It was defined as Equation 1:

(1) $$BIC(G,D)=\text{log }P(D|G)-\frac{\left|\theta \right|}{2}\text{log }(n)$$

Where *G* denoted the graph, *D* the data, *θ* the set of free parameters, and *n* the sample size. A higher BIC score indicated a better trade-off between model fit and complexity, favouring simpler models that explained the data effectively.

5. Log-Likelihood (LL): The LL score measured how well the learnt graphs fitted the observed data. A higher log-likelihood value indicated better fit; however, model fit naturally improved with each additional edge. Therefore, the BIC score was used to balance model fit against complexity.
6. F1: The F1 score evaluated the balance between precision and recall when assessing the accuracy of predicted edges. It was mathematically expressed as Equation 2:

(2) $$F1=\frac{2\cdot RP}{R+P}$$

where *R* represented recall (the proportion of true positive connections) and *P* represented precision (the proportion of true positive connections among all predicted connections).

7. Balanced Scoring Function (BSF): Unlike SHD, which focused solely on false positives (FP) and false negatives (FN), and F1, which did not account for true negatives (TN), the BSF metric evaluated graph similarity by considering all four parameters of the confusion matrix. The BSF score ranged from -1 to 1. It returned a balanced evaluation, where both empty and fully connected networks produced a score of 0, reflecting equal ignorance. The BSF score was calculated as Equation 3:

(3) $$BSF\;=0.5\;(\frac{TP}{a}\;+\;\frac{TN}{i}\;-\;\frac{FP}{i}-\;\frac{FN}{a})$$

where *TP* was the number of true positives (correctly identified edges), *TN* was the number of true negatives (correctly identified absent edges), *FP* was the number of false positives (incorrectly identified edges), *FN* was the number of false negatives (missed edges), *a* was the number of edges in the ground truth, and *i* was the number of independencies in the ground truth, calculated as $i=\frac{\left|V|(|V|-1\right)}{2}$ where |*V* |, was the number of variables.

## 3 Results and discussion

The evaluation began in subsection 3.1 by investigating the similarities between graphical structures learnt from the data and the knowledge graph. Subsection 3.2 subsequently examined differences in the effects of interventions produced by some of these structures.

Finally, subsection 3.3 identified key variables that significantly influenced NICU admission and maternal trauma.

**Figure 4:**
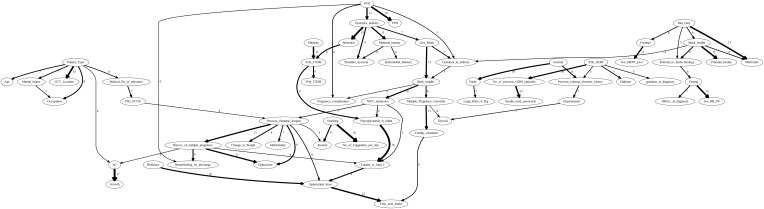
The model-averaging graph depicting the frequency of directed edges obtained from 20 structure learning algorithms. Edges appearing less than four times across the 20 learnt outputs are excluded. The graph contains 75 directed edges across 62 nodes.

The model-averaged graph, shown in **Figure 4**, represented aggregated insights from 20 structure learning algorithms. Comprising 75 directed edges across 62 nodes, the graph excluded edges that appeared in fewer than four graphs, highlighting substantial disagreement across methods. This sparsity reflected the challenges inherent to the dataset, which contained only 1808 samples. The relatively small sample size likely limited the identifiability of causal relationships, contributing to variability across algorithmic outputs and reducing the reliability of inferred structures. Nevertheless, the absence of undirected edges in the model-averaged graph enhanced the clarity of the representation, offering a more refined and interpretable depiction of causal relationships within the GDM cohort.

### 3.1 Structure learning performance

**Table 3** provides a detailed comparison of the performance metrics for the various structure learning algorithms employed in this study, with reference to the metrics described in subsection 2.4. The results revealed substantial discrepancies between the data-driven structures and the knowledge-based reference graph, reported with 95% confidence intervals. For instance, SHD values ranged from 32.2 (LiNGAM) to 124.9 (GIES) with relatively lower scores for GS (36.01) and MMHC (40.30) attributed primarily to the sparsity of their inferred graphs rather than genuine alignment with the knowledge graph. Even the best-performing SHD scores indicated significant structural mismatches, highlighting notable inconsistencies in the learnt causal structures. Low F1 scores, peaking at 0.16 (MMHC), indicated that none of the algorithms effectively recovered causal edges as identified by 465 clinical expertise and GPT-4, while GS and LiNGAM failed to recover any true causal edges. Negative BSF scores across all algorithms reflected the difficulty of reconciling data-driven inferences with the knowledge graph. Models such as SaiyanH and GES achieved a better balance between data fit and complexity, as indicated by LL and BIC scores, while overparameterised models like NOTEARS and GIES demonstrated poor generalisability. Sparse models, such as MMHC and RSMAX2, exhibited greater generalisability but produced fragmented structures, limiting their practical utility.

**Table 3:** Comparison of structure learning algorithms based on Structural Hamming Distance (SHD), F1 score, Balanced Scoring Function (BSF) with reference to the knowledge graph, Log-Likelihood (LL), Bayesian Information Criterion (BIC), number of free parameters, and number of independent graphical fragments.

Algorithm	SHD	F1	BSF	LL	BIC	Free params	Fragments
SaiyanH	57.3 [47.0, 68.0]	0.08 [0.03, 0.13]	-0.40 [-0.45, -0.34]	-88.8k	-92.6k	698	1
HC	55.4 [45.0, 67.0]	0.12 [0.06, 0.18]	-0.37 [-0.43, -0.30]	-88.5k	-92.3k	708	2
TABU	56.4 [46.0, 68.0]	0.10 [0.03, 0.15]	-0.39 [-0.45, -0.32]	-88.5k	-92.3k	704	2
H2PC	47.6 [39.0, 57.0]	0.11 [0.04, 0.15]	-0.40 [-0.46, -0.35]	-93.1k	-95.6k	465	26
MMHC	40.3 [32.0, 48.0]	0.16 [0.11, 0.20]	-0.38 [-0.42, -0.31]	-95.7k	-97.9k	412	34
MAHC	47.9 [38.0, 58.0]	0.13 [0.07, 0.20]	-0.37 [-0.43, -0.30]	-89.6k	-92.9k	610	9
GES	58.3 [48.0, 70.0]	0.11 [0.06, 0.17]	-0.37 [-0.43, -0.30]	-88.4k	-92.3k	724	2
PCSTABLE	43.2 [35.0, 52.0]	0.02 [0.00, 0.04]	-0.47 [-0.49, -0.44]	-94.3k	-98.0k	682	33
IAMB	54.7 [45.0, 64.0]	0.02 [0.00, 0.04]	-0.46 [-0.48, -0.43]	-93.7k	-98.3k	841	25
fastIAMB	51.4 [41.0, 61.0]	0.06 [0.03, 0.07]	-0.44 [-0.46, -0.42]	-93.9k	-97.8k	723	26
RSMAX2	41.4 [33.0, 51.0]	0.16 [0.11, 0.20]	-0.38 [-0.42, -0.31]	-94.1k	-96.4k	420	33
InterIamb	56.3 [46.0, 66.0]	0.02 [0.00, 0.03]	-0.46 [-0.48, -0.43]	-93.5k	-98.1k	855	23
IAMBFDR	53.4 [43.0, 63.0]	0.06 [0.03, 0.07]	-0.44 [-0.46, -0.39]	-94.3k	-98.5k	777	24
MMPC	44.8 [37.0, 53.0]	0.06 [0.04, 0.08]	-0.45 [-0.46, -0.42]	-93.1k	-96.3k	593	24
GS	36.0 [29.0, 43.0]	0.00 [0.00, 0.00]	-0.49 [-0.49, -0.49]	-101.3k	-104.1k	513	45
LiNGAM	32.2 [26.0, 38.0]	0.00 [0.00, 0.00]	-0.49 [-0.49, -0.49]	-103.7k	-105.9k	410	44
NOTEARS	100.4 [73.0, 126.0]	0.03 [0.00, 0.06]	-0.37 [-0.41, -0.31]	-91.7k	-28.9M	5.3M	4
GIES	124.9 [104.0, 145.0]	0.04 [0.00, 0.07]	-0.35 [-0.41, -0.28]	-1.8e43	-1.8e43	6.5M	1
FGES	78.6 [66.0, 92.0]	0.09 [0.03, 0.14]	-0.36 [-0.43, -0.29]	-86.5k	-97.0k	1,946	1
IMAGES	78.6 [66.0, 92.0]	0.09 [0.03, 0.14]	-0.36 [-0.43, -0.29]	-86.5k	-97.0k	1,946	1
Model Average	62.3 [50.0, 74.0]	0.11 [0.03, 0.17]	-0.37 [-0.44, -0.30]	-87.6k	-97.7k	1,853	1

The model-averaged graph provided a consensus-driven representation that prioritised frequently identified edges while excluding inconsistent ones. Its SHD of 62.3 indicated improved alignment with the knowledge graph, successfully capturing both strongly and moderately supported relationships. With an F1 score of 0.11 and a BSF of –0.37, the model-averaged graph achieved a better balance between precision and recall than individual models characterised by extreme sparsity or density. Its moderate complexity (1,853 free parameters) and the presence of a single graphical fragment enhanced interpretability, while its BIC score (-97.0k) reflected an acceptable trade-off between model fit and parsimony. These findings highlighted the value of model averaging in mitigating variability across algorithms, offering a stable and interpretable framework for representing causal structures in high-dimensional, sample-constrained datasets.

**Figure 5:**
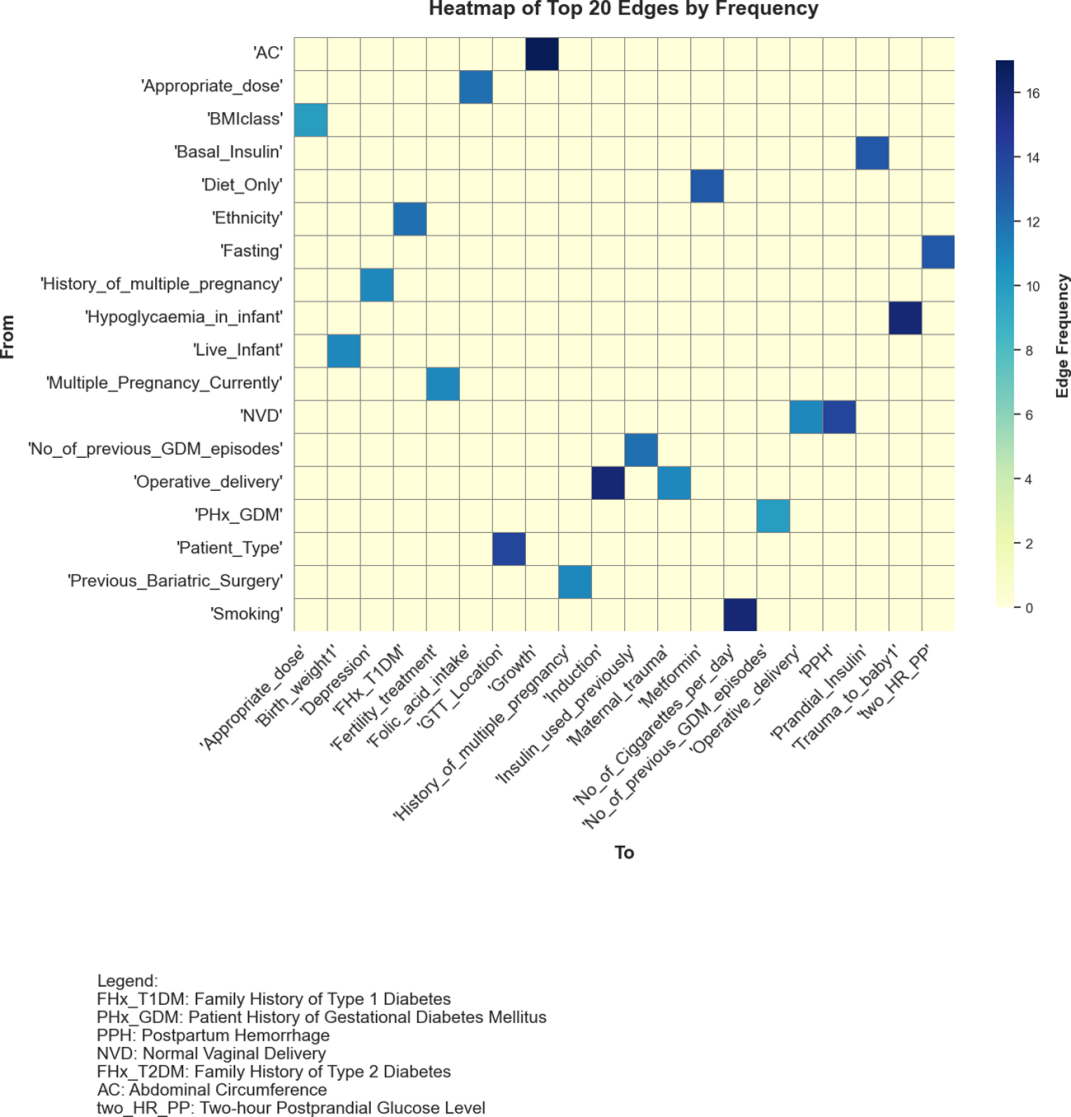
Heatmap showcasing the top 20 most frequently identified edges across graphs learnt from 20 algorithms. The variables on both axes represent different features, with darker blue shades indicating edges that were more consistently discovered by the algorithms.

**Figure 5** presents a heatmap of the top 20 most frequently identified edges across the graphs generated by the 20 structure learning algorithms, demonstrating considerable variability in algorithmic outputs. The low level of consensus, with only a few edges consistently appearing across multiple algorithms, highlighted the inherent challenges of structure learning in datasets with high dimensionality and limited sample sizes.

Edges such as Abdominal Circumference (AC) → Growth and Smoking →No_of_Cigarettes_per_day, identified by 17 and 16 algorithms respectively, indicated strong agreement amongst the algorithms. The smoking-related edge aligned with established associations in the data, while AC → Growth reflected a data-driven relationship that warranted further investigation to establish its validity. Moderately frequent edges, appearing in 8 to 13 algorithms, including Diet_Only → Metformin and Basal_Insulin → Prandial_Insulin, suggested potential relationships but showed varying levels of agreement with domain knowledge. These edges may reflect statistical associations rather than direct causal links, necessitating careful evaluation to determine their significance.

Rarely identified edges, such as Diabetes → Insulin_used_previously and Ethnicity → Diabetes, which appeared in only one model, reflected the variability in algorithmic outputs. These edges may have captured dataset-specific nuances or may simply have arisen as statistical artefacts. Their presence highlighted the need for further analysis to distinguish meaningful findings from spurious patterns.

Overall, the findings revealed the sensitivity of structure learning algorithms to dataset characteristics and underlying modelling assumptions, resulting in substantial variability in inferred causal structures. The limited agreement across algorithms underscored the importance of model averaging as a strategy to enhance the reliability of identified relationships. Frequent edges offered strong candidates for validation, while rare edges provided opportunities for exploratory analyses and hypothesis generation.

### 3.2 Causal inference and intervention

The goal of the interventional analysis was to forecast the impact of potential interventions before their application in real-world clinical settings. Given that the dataset exclusively included patients diagnosed with GDM, the primary aim was to understand the influence of modifiable factors on maternal and neonatal outcomes. Specifically, the aim was to explore how interventions could affect maternal outcomes, such as maternal trauma, and neonatal outcomes, including NICU admission. Identifying and assessing these key interventions through simulation provided actionable insights aimed at improving health outcomes for both mothers and newborns.

To quantify the effects of each intervention, outcome probabilities were estimated under both intervention and non-intervention scenarios using the *do*-operator. The resulting differences, calculated as P(Y|do(X=1))−P(Y|do(X=0)) , represent absolute risk differences. These values are expressed in percentage point terms. While this approach offers a clear measure of interventional impact, confidence intervals for these estimates could not be reported, asthe GeNIe software used does not support uncertainty estimation for interventional queries.

Using both the model-averaged graph and the knowledge graph, the potential effects of various interventions were evaluated. While the model-averaged graph provided actionable insights for multiple outcomes, including NICU admission rates and pregnancy complications, the knowledge graph revealed significant limitations in its ability to suggest interventions beyond NICU admission rates. Specifically, for NICU admission rates, the knowledge graph demonstrated a comparable pattern, albeit with slight variations in the magnitude of changes across birth weight categories. However, for other variables, such as pregnancy complications, no meaningful interventional insights could be derived from the knowledge graph.

We examined the orientation frequencies of the causal edges supporting each intervention within the model-averaged graph, derived from 20 structure learning algorithms. Interventions such as NICU admission and birth weight (frequency = 10) and previous bariatric surgery and maternal weight gain (8) were associated with relatively high majority-vote counts, indicating greater stability in edge orientation. In contrast, associations such as pregnancy complications and birth weight (4), history of multiple pregnancies and neonatal trauma (4), patient history of GDM and gestation at diagnosis (5), and fasting glucose and diet-only intervention (6) exhibited lower orientation frequencies, suggesting greater uncertainty in causal direction. No direct edge was identified between parity and patient history of GDM.

A full sensitivity analysis is presented in **Table 4**, in which progressively stricter majority-vote thresholds were applied to the model-averaged graph to evaluate the stability of candidate intervention edges. The table demonstrates how algorithmic support for individual causal relationships attenuates as the frequency cut-off is increased from four to twelve out of twenty algorithms. The association between birth weight and NICU admission showed the greatest robustness, persisting up to the 10-vote threshold, whereas the link between previous bariatric surgery and change in maternal weight was maintained only up to eight votes. By contrast, weaker relationships, including birth weight with pregnancy complications and history of multiple pregnancies with neonatal trauma, disappeared once the threshold was raised beyond the minimum. Notably, no direct edge was recovered between parity and prior history of GDM at any cut-off, indicating a lack of reproducible evidence across algorithms for this relationship.

**Figure 6:**
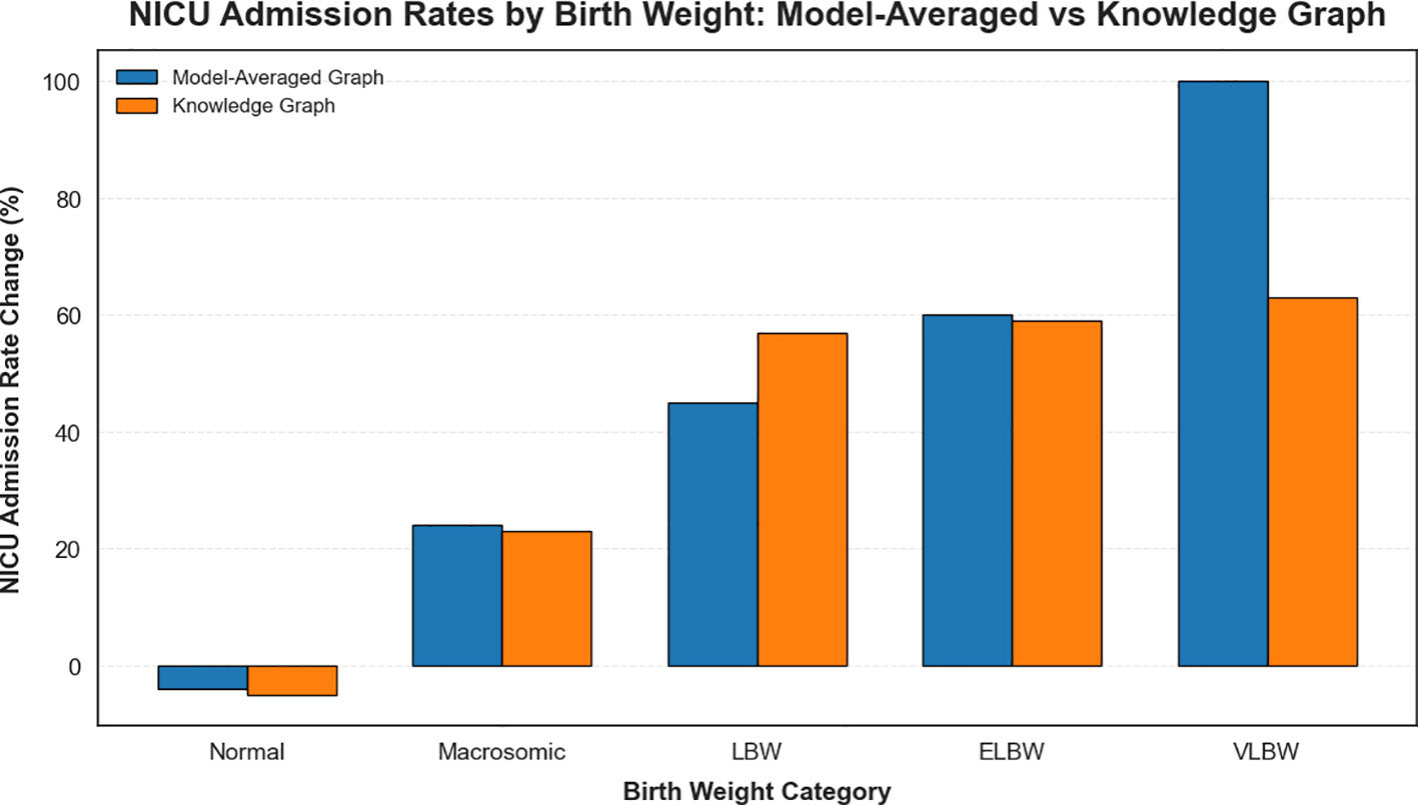
Comparison of NICU admission rates by birth weight category derived from the model-averaged graph and the knowledge graph.

**Figure 6** compares the NICU admission rates derived from the model-averaged graph and the knowledge graph, highlighting the limited scope of interventions available in the latter.

**Figure 7:**
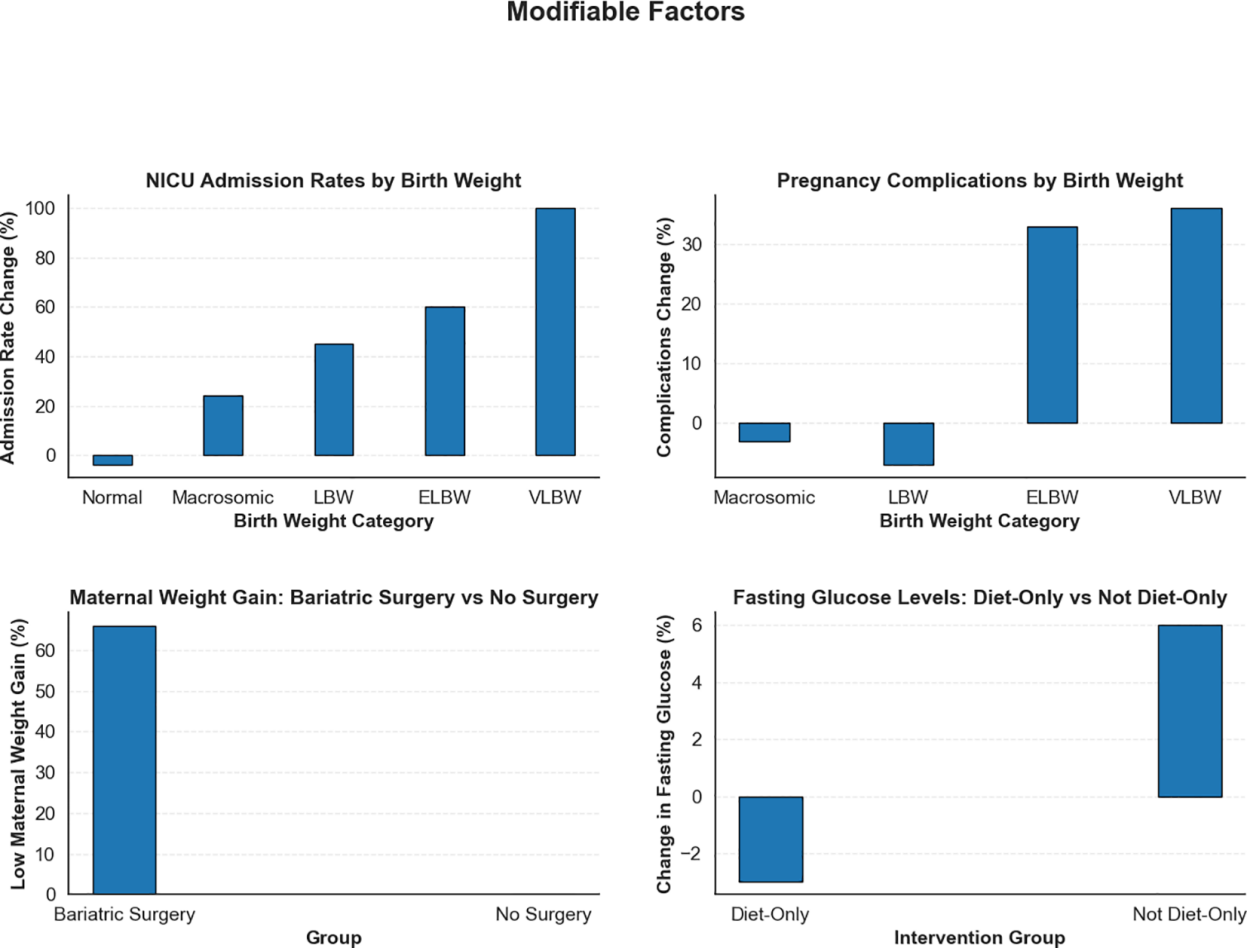
Impact of modifiable risk factors on maternal and neonatal outcomes in GDM patients. This figure illustrates the effect of various modifiable factors on critical maternal and neonatal outcomes.

To build upon these findings, further analysis examined the outcomes of various interventions on maternal and neonatal health in GDM patients. This stage leveraged the strengths of the model-averaged results to provide a more comprehensive understanding of modifiable risk factors (**Figure 7**).

#### 3.2.1 Previous bariatric surgery and maternal weight gain

Previous bariatric surgery significantly influenced maternal weight gain during pregnancy. Women with a history of bariatric surgery demonstrated a 66% increase in low maternal weight gain compared to those without such a history. Although bariatric surgery provides long-term benefits for obesity management, it may disrupt metabolic processes during pregnancy. These findings suggest that women with prior bariatric surgery require close monitoring and tailored nutritional support to optimise maternal and fetal outcomes (52).

#### 3.2.2 Fasting glucose and diet-only intervention

The impact of a diet-only intervention on fasting glucose levels after delivery in GDM patients was evaluated. Patients were classified as either “Diet-Only” or “Not Diet-Only” based on whether dietary management constituted their primary treatment during pregnancy. The analysis showed that those managed with diet alone experienced a 3% reduction in elevated fasting glucose levels, whereas patients requiring additional interventions exhibited a 6% increase in at-risk fasting glucose levels postpartum. This trend likely reflected differences in the severity of hyperglycaemia during pregnancy, as individuals treated solely with diet may have had milder forms of GDM compared to those needing pharmacological therapies such as insulin or metformin. Consequently, women who required pharmacological treatment appeared to represent a higher-risk group, warranting closer postpartum monitoring. These findings underscore the importance of tailoring postnatal care based on the severity of GDM and the type of antenatal management received.

##### 3.2.1.1 Non-modifiable risk factors and their influence on maternal and neonatal outcomes

**Figure 8:**
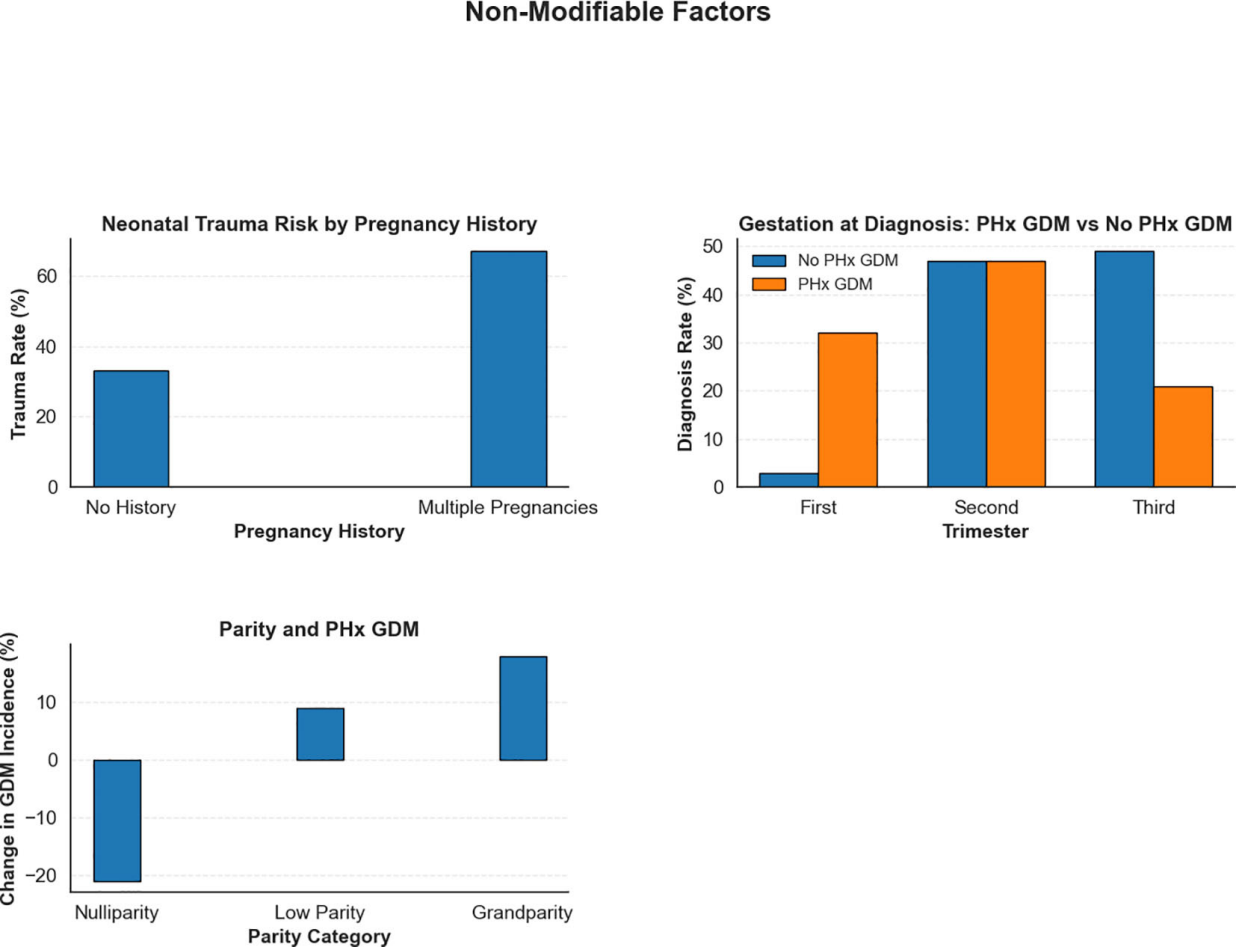
Impact of non-modifiable risk factors on maternal and neonatal outcomes in GDM patients. This figure emphasises the influence of intrinsic risk factors.

In addition to the modifiable risk factors analysed in this study, the role of non-modifiable factors in shaping maternal and neonatal outcomes among GDM patients was also explored. Although these factors cannot be altered through clinical interventions, their identification remains crucial for informing personalised care strategies and improving risk stratification. The effects of key non-modifiable factors were evaluated using the clinical dataset and are described below, with corresponding visualisations presented in **Figure 8**.

###### 3.2.1.1.1 NICU admission and birth weight

Birth weight significantly influenced NICU admissions. For normal birth weight infants, NICU admissions decreased by 4%, as expected due to their generally lower need for intensive care. However, macrosomic infants exhibited a 24% increase in NICU admissions, reflecting the heightened risks associated with larger birth weights. Low birth weight (LBW) infants experienced a 45% rise in NICU admissions, while extremely low (ELBW) and very low birth weight (VLBW) infants reached admission rates of 75%and 100%, respectively, indicating the need for urgent intervention in these cases (53).

###### 3.2.1.1.2 Pregnancy complications and birth weight

Birth weight also had a substantial impact on pregnancy complications. For normal birth weight infants, pregnancy complications were relatively uncommon, with a 3% reduction. Macrosomia was linked to a modest 7% decrease in complications. However, infants with low birth weight were at significantly greater risk, with pregnancy complications increasing by 33% in LBW cases and by 36% in VLBW cases, highlighting the heightened vulnerability of underweight neonates to adverse outcomes (53).

###### 3.2.1.1.3 History of multiple pregnancies and neonatal trauma

A history of multiple pregnancies significantly increased the risk for neonatal trauma. In pregnancies without this history, the observed proportion of neonatal trauma cases was 33%. This percentage is presented for comparative purposes only and doesnot imply that such a rate is clinically acceptable; any occurrence of neonatal trauma is undesirable. In patients with a history of multiple pregnancies, the trauma rate rose to 67%. These findings underscore the need for specialised obstetric care forwomen with multiple previous pregnancies, including careful labour management and consideration of elective caesarean delivery, to help reduce adverse outcomes associated with multiple pregnancies.

###### 3.2.1.1.4 Patient history of GDM and gestation at diagnosis

The effect of a prior history of GDM (PHx GDM) on the timing of diagnosis in subsequent pregnancies was assessed. Among women without prior GDM, most were diagnosed during the second (47%) and third trimesters (49%, a 6% increase), with only 3% diagnosed in the first trimester (a 6% decrease). In contrast, women with a previous history of GDM showed a notable shift towards earlier detection: first-trimester diagnoses rose to 32% (an increase of 23%), while third-trimester diagnoses decreased to 21% (a 22% reduction). These results emphasised the importance of early screening for women with prior GDM, as early diagnosis enables timely intervention and better glycaemic control, thereby reducing the risk of complications (54).

###### 3.2.1.1.5 Parity and patient history of GDM (PHx GDM)

To understand the impact of parity on GDM risk, the presence of a history of GDM (PHx GDM) across different parity categories was assessed: nulliparity (no previous pregnancies), low parity (one or two previous pregnancies), and grandparity (three or more previous pregnancies). Our analysis revealed a significant relationship between parity and GDM risk. For nulliparous women, the intervention resulted in a notable 21% decrease in the incidence of GDM, while low parity women had a 9% increase. Grandparous women exhibited the highest risk, with an 18% increase in PHx GDM incidence. These findings highlighted the importance of parity as a risk factor for GDM. Close monitoring of glucose levels and proactive management of GDM is especially critical for women with high parity. Targeted prenatal care, including frequent glucose monitoring and early interventions, could help mitigate the risks associated with grandparity and improve maternal and neonatal outcomes (55).

The model-averaged causal structure highlighted several intervention opportunities that closely align with established clinical practices. While many of the relationships identified by the model are already recognised in clinical settings, clinicians emphasised that the ability to quantify these relationships added significant value to decision-making processes. Quantitative insights, such as the magnitude of impact from factors like dietary management, glucose monitoring, and specialised care for high-risk subgroups (e.g., patients with a history of GDM), provided clearer guidance for prioritising interventions. By offering a measurable understanding of how modifiable factors influenced maternal and neonatal outcomes, the model supported more targeted, patient-specific strategies, ultimately enhancing the effectiveness of clinical care.

Clinicians further highlighted that such quantitative insights could improve resource allocation, allowing healthcare providers to identify patients most likely to benefit from intensive management or early intervention. They also recognised the model’s potential to support patient education by translating complex data into actionable metrics, thereby helping individuals better understand how specific lifestyle modifications and treatment options may influence their health outcomes. The combination of quantifiable evidence and clinical relevance underscored the model’s utility in guiding both population-level strategies and personalised care plans.

### 3.3 Sensitivity analysis

Sensitivity analysis evaluated how the probability distribution of a node responded to modifications in its parent or ancestor nodes. A node displaying high sensitivity exhibited substantial shifts in its posterior distribution even with minor changes to the parameters of its Conditional Probability Table (CPT), indicating a strong dependency on upstream factors. Conversely, low sensitivity suggested that even significant changes in CPT parameters led to only minimal alterations in the node’s posterior distribution, reflecting a weaker dependency.

In this study, sensitivity analysis focused on two key clinical outcomes: NICU admission and maternal trauma. GeNIe BN software (49) was employed to quantify the impact of variations in the parameters of parent and ancestor nodes on outcomes in both the model-averaged network and the knowledge graph. This analysis offered valuable insights into the strength and nature of dependencies within the model, identifying the factors that most significantly influenced the observed outcomes.

**Figure 9:**
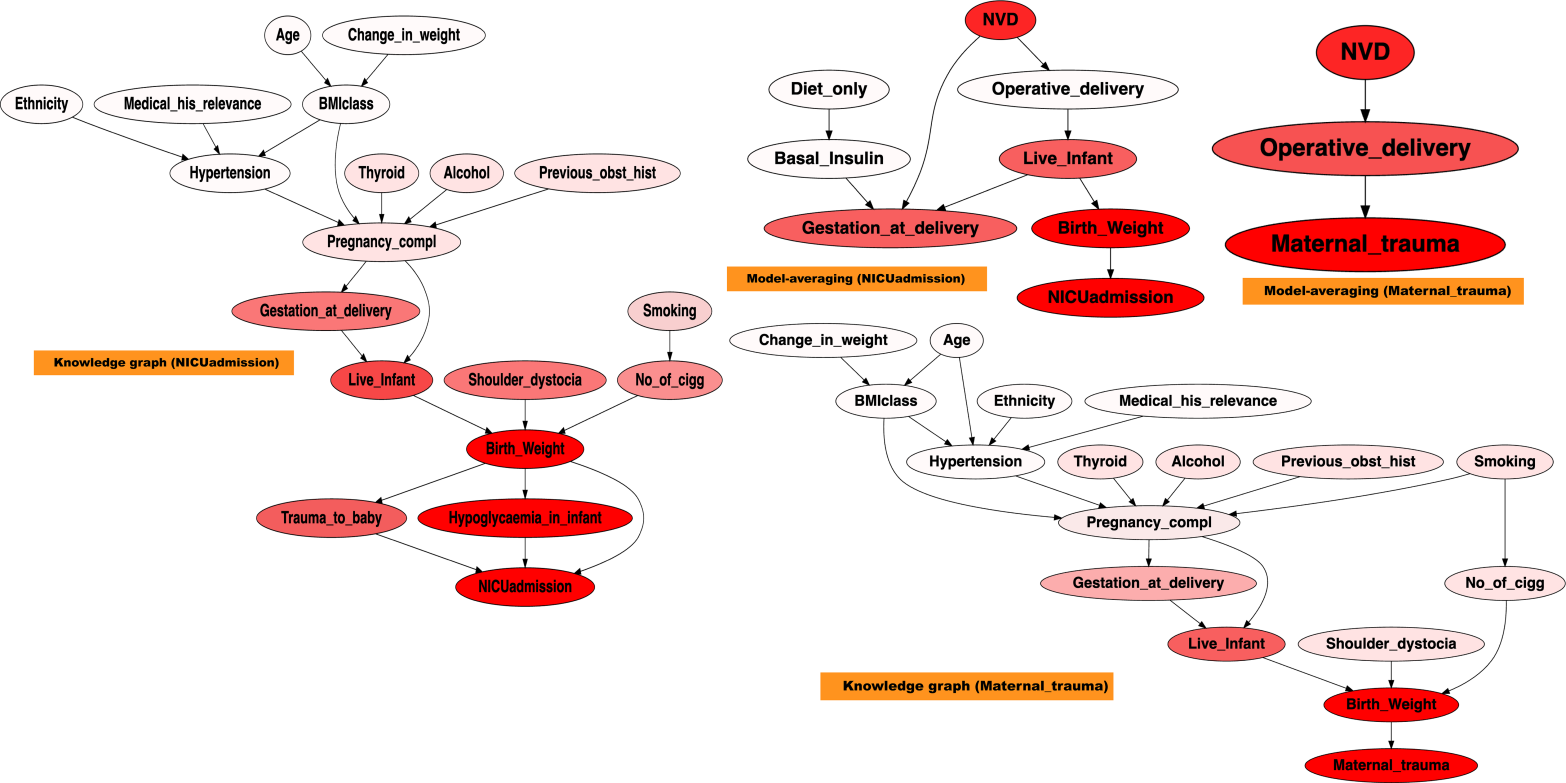
Sensitivity analysis on NICUadmission and maternal trauma, across the modelaverage graph, and the knowledge graph. A stronger red colour indicates higher sensitivity.

**Figure 9** presents the sensitivity analysis for NICUadmission and Maternal_trauma, based on graphs derived from both domain knowledge and the model-averaged approach. For clarity, only the relevant graph fragments are shown. Nodes are highlighted in red, with darker shades indicating stronger sensitivity.

**Table 4:** Sensitivity of intervention edges to stricter majority-vote cut-offs in the modelaveraged graph (20 algorithms total).

Intervention edge	≥4	≥6	≥8	≥10	≥12
Previous Bariatric Surgery → Change in Weight	Yes	Yes	Yes	No	No
Fasting → Diet-Only	Yes	Yes	No	No	No
Birth Weight → NICU Admission	Yes	Yes	Yes	Yes	No
Birth Weight → Pregnancy Complications	Yes	No	No	No	No
History of Multiple Pregnancies → Neonatal Trauma	Yes	No	No	No	No
Prior History of GDM → Gestation at Diagnosis	Yes	No	No	No	No
Parity, Prior History of GDM (no direct edge)	No	No	No	No	No

“Yes” indicates the edge is retained at the specified cut-off.

The sensitivity analysis highlighted the diverse and interacting determinants contributing to NICU admission and Maternal_trauma. In the knowledge graph, NICUadmission was sensitive to Live_infant,Trauma_to_baby, Birth_weight, and Hypoglycaemia_in_infant. Maternal_trauma demonstrated sensitivity to Birth_weight and Live_infant. In contrast, the model-averaged graph revealed that NICUadmission was influenced by Birth_weight, Gestation_at_delivery, and Live_infant, while maternal trauma showed sensitivity to Birth_weight, Operative_delivery, and Normal Vaginal Delivery (NVD).

NICUadmission appeared highly sensitive to variations in birth weight, with infants outside the normal range being more likely to require intensive care, consistent with findings from previous studies (56, 57). In addition, both gestational age at delivery and the occurrence of neonatal hypoglycaemia significantly influenced NICU requirements, reinforcing their roles as key predictors of adverse neonatal outcomes (57).

Maternal_trauma demonstrated notable sensitivity to variations in birth weight, operative delivery, and NVD. Studies have consistently shown that deviations in Birth_weight and mode of delivery are closely linked to the risk of Maternal_trauma, highlighting their relevance in predicting maternal complications (58).

Overall, the sensitivity analysis revealed a complex interaction among factors influencing NICUadmission and Maternal_trauma, with Birth_weight standing out as the most important determinant. These results aligned closely with the established literature, further validating the importance of early risk identification and targeted clinical interventions in the management of GDM-related outcomes.

## 4 Conclusion

This study applied structure learning techniques to model causal relationships in GDM using a clinically curated dataset from a large Irish maternity unit. A knowledge graph was developed by combining GPT-4 outputs, a targeted literature review, and clinical expert input, ensuring clinical relevance and validity. To explore data-driven relationships, 20 structure learning algorithms were employed. Given the variability in their outputs, a model-averaging approach was used to generate a consensus causal network that aligned more closely with expert knowledge and improved interpretability.

The model-averaged causal graph enabled the identification of key factors influencing maternal and neonatal outcomes. Birth weight emerged as a key determinant of NICU admissions, with both macrosomia and low birth weight linked to increased risk. Clinicians involved in the study noted that while these risks were already recognised in practice, the ability to quantify their magnitude strengthened clinical confidence in prioritising targeted interventions. Although birth weight itself is not directly modifiable, the analysis highlights the significance of addressing upstream determinants of fetal growth during pregnancy. Other clinically relevant variables, including timing of GDM diagnosis, prior bariatric surgery, and history of multiple pregnancies, also shaped risk profiles. Sensitivity analysis reinforced these findings, identifying birth weight, gestational age, and delivery mode as major contributors to NICU admission and maternal trauma.

Compared to existing GDM risk scores and prediction models, which often rely on regression-based or machine learning classifiers trained on a limited set of predefined variables, our approach offers a complementary and more interpretable perspective. Traditional models typically support binary risk stratification (e.g., high vs. low GDM risk) and provide limited insight into the underlying causal mechanisms. In contrast, causal Bayesian networks offer a structured framework that captures both the directionality and conditional dependencies among variables. This enables simulation of counterfactual scenarios and identification of upstream, modifiable factors that may influence clinical outcomes. For instance, rather than merely predicting NICU admission, our model facilitates exploration of how interventions targeting gestational weight gain or treatment timing could affect birth weight and downstream neonatal complications. To our knowledge, few studies have applied causal modelling of this scale and scope within the context of GDM, underscoring the novelty and translational value of our framework.

Despite these contributions, several limitations should be acknowledged. The analysis was based on a single-centre dataset, which may limit the generalisability of findings to other populations or care settings. High rates of missing data in certain variables introduced uncertainty, and although a structured approach was used to address this, the assumption of missingness at random remains a potential source of bias. Causal relationships were inferred from observational data using structure learning techniques, which, in the absence of experimental validation, remain susceptible to unmeasured confounding. In cases where algorithms produced partially directed graphs, forced edge orientations were applied to ensure acyclicity, potentially introducing directionality artefacts. The initial knowledge graph incorporated suggestions from GPT-4 to guide structure construction; while these outputs were reviewed and refined by clinical experts, they do not substitute for full consensus among domain specialists. Finally, no external or temporal validation was performed, owing to the unavailability of comparable datasets and the absence of time-specific modelling, which limits the evaluation of model stability across settings or time periods. Future research should focus on validating these findings in larger, more diverse cohorts to enhance their clinical applicability and support broader implementation of personalised care strategies in GDM management.

## Data Availability

The datasets presented in this article are not readily available because the dataset used in this study, derived from patient records at CUMH, is not publicly available due to ethical and confidentiality restrictions associated with patient data. However, the graphs and model outputs generated during the analysis are available in the repository (59). Requests to access the datasets should be directed to http://bayesian-ai.eecs.qmul.ac.uk/bayesys/.
